# The splicing FK506-binding protein-51 isoform plays a role in glioblastoma resistance through programmed cell death ligand-1 expression regulation

**DOI:** 10.1038/s41420-019-0216-0

**Published:** 2019-09-24

**Authors:** Paolo D’Arrigo, Marina Digregorio, Simona Romano, Martina Tufano, Anna Rea, Felix Hausch, Matthias Dedobbeleer, Vincenza Vigorito, Salvatore Russo, Michael Bauder, Bernard Rogister, Maria Fiammetta Romano

**Affiliations:** 1 Dipartimento di Medicina Molecolare e Biotecnologie Mediche, Università di Napoli Federico II, Napoli, Italy; 2 GIGA-Neurosciences, Faculté de Médecine, Liège Université de Liège, Liège, Belgium; 3 Technical University Darmstadt Institute of Organic Chemistry and Biochemistry, Darmstadt, Germany

**Keywords:** Apoptosis, CNS cancer

## Abstract

Gliomas aberrantly express programmed cell death ligand-1 (PD-L1), which has a pivotal role in immunoevasion. The splicing isoform of *FKBP5*, termed FKBP51s, is a PD-L1 foldase, assisting the immune checkpoint molecule in maturation and expression on the plasma membrane. The concept that PD-L1 supports tumor-intrinsic properties is increasingly emerging. The aim of the present work was to confirm the pro-tumoral effect of PD-L1 on human glioma cell survival, stemness capacity and resistance, and to address the issue of whether, by targeting its foldase either chemically or by silencing, the aggressive tumor features could be attenuated. PD-L1-depleted glioma cells have a reduced threshold for apoptosis, while PD-L1 forced expression increases resistance. Similar results were obtained with FKBP51s modulation. The ability of PD-L1 to counteract cell death was hampered by FKBP51s silencing. PD-L1 expression was particularly high in glioma cells with a cancer-stem-cell profile. Moreover, PD-L1 sustained the spheroid formation capability of glioma cells. Targeting of FKBP51s by small-interfering RNA (siRNA) or the specific inhibitor SAFit2, reduced the number of formed spheroids, along with PD-L1 expression. Finally, in an orthotopic mouse model of glioblastoma, daily treatment with SAFit2 significantly reduced tumor PD-L1 expression, and tumor growth. In treated mice, caspase-3 activation and reduced vimentin expression were observed in excised tumors. In conclusion, targeting of FKBP51s hampers PD-L1 and its pro-tumoral properties, thereby affecting the self-renewal and growth capacities of glioblastoma cells in vitro and in vivo.

## Introduction

Glioblastoma multiforme (GBM) is the most common, but also the most dangerous and aggressive form of primary brain cancer, for which no contemporary treatments are curative. Gliomas, indeed, express several co-inhibitory molecules that negatively regulate immune system functions^1–4^, among which programmed cell death ligand-1 (PD-L1) is one of the most represented and studied^3,4^. The interaction of PD-L1 with its counter-receptor programmed cell death-1 (PD-1) expressed by activated T lymphocytes, inhibits T-cell activation and proliferation, thus contributing to cancer immune evasion^5^. So far, anti-PD-1 treatment has shown a therapeutic efficacy limited to selected subsets of glioma patients^6^. Several studies are in progress, especially exploring the combination of anti-PD-1 antibodies with chemotherapies or targeted therapies^6^. It is worth noting that PD-L1 not only binds to PD-1 but also competes with CD28-to-CD80 binding^7^, suggesting that a PD-1 blockade may sometimes be insufficient to prevent signals emanating from PD-L1 receptors.

Recent studies show that, in addition to its immunomodulatory function, PD-L1 conveys a survival signal to the cancer cell and sustains tumor aggressiveness^6^. Azuma et al.^8^ found that PD-L1, upon ligand stimulation, acts as a receptor that transduces pro-survival signals that enhance the threshold for apoptosis induced by both immune effectors and proapoptotic drugs. Zheng et al.^9^ found that PD-L1 sustains the self-renewal capability of malignant melanoma-initiating cells. Most recently, Qiu et al.^10^ demonstrated that PD-L1 binds to Ras and activates the Ras/Erk pathway. Via this mechanism, PD-L1 promotes the epithelial-to-mesenchymal transition (EMT) and GBM cell migration.

Akt supports aberrant PD-L1 expression in glioma cells, through a translational mechanism favouring the assembly of a polyribosome complex that allows better entrance of the PD-L1 transcript into polysomes^11^. PD-L1 glycosylation is an essential element influencing protein stability^12^. Most recently, in glioma, the splicing isoform of the FK506-binding protein-51 (FKBP51s)^13^ was found to serve as a PD-L1 co-chaperone, assisting in protein glycosylation^14^. PD-L1 post-translational modifications have been thoroughly reviewed by Hsu et al.^15^. According to Jiang et al.^16^ and the Oncomine database (www.oncomine.org), FKBP51 is among the top 10% of the most highly expressed genes in GBM. The FKBP51 protein structure includes a C-terminal TPR three-tandem-repeat domain responsible for protein-protein interaction, and two N-terminal FK domains, of which the one with most N terminals exhibits peptidyl-prolyl cis-trans isomerase (PPIase) activity^17,18^. FKBP51s is generated by alternative splicing of *FKBP5* pre-mRNA^13^, which causes a frameshift with a premature stop codon, leading to a distinct C-terminus, compared to the canonical isoform. FKBP51s retains the PPIase activity but loses the TPR domain. An IHC study on 29 GBM specimens showed that FKBP51s is broadly expressed in this tumor, albeit with different proportion/intensity scores, with nuclear and/or cytoplasmic localization^14^. Biochemical evaluation of glioma cell lines showed that naive PD-L1 is complexed with FKBP51s in the endoplasmic reticulum, whereas the glycosylated form was detected in the Golgi apparatus^14^. FKBP51s knockdown severely reduced the level of glycosylated PD-L1, whether constitutively expressed or induced by ionizing radiation^14^. The essential role of the PPIase activity in PD-L1 protein maturation was confirmed by use of a selective inhibitor of this catalytic function, i.e., SAFit2, which produced similar results to those found in FKBP51s silencing^14^.

The present study confirms that PD-L1 exerts important tumor-intrinsic properties in GBM. In particular, we show that PD-L1 sustains cell survival, resistance and stemness capability. PD-L1 expression was highest in GBM cancer-initiating cells, due to a post-transcriptional regulatory effect involving FKBP51s. Targeting of FKBP51s by gene silencing or via the selective inhibitor SAFit2, downmodulated PD-L1 expression and inhibited spheroid formation when GBM cancer-initiating cells were cultured under non-adherent conditions. In an orthotopic GBM mouse model, SAFit2 showed an anti-tumor effect, as assessed by reductions in tumor volumes, caspase activation and attenuated expression levels of PD-L1 and the mesenchymal marker vimentin.

## Results

### PD-L1 promotes apoptosis resistance

**Fig. 1:**
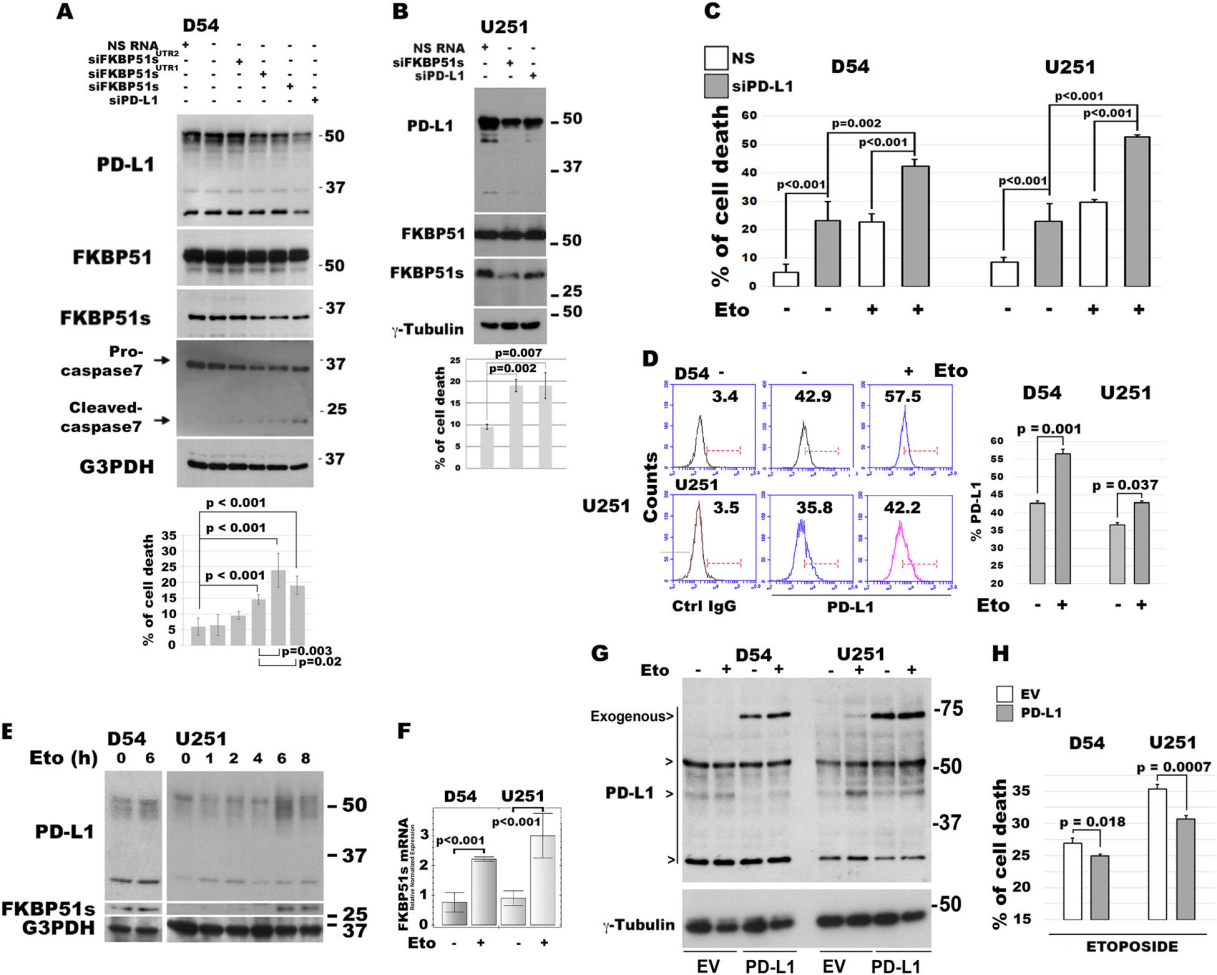
PD-L1 regulates glioma cell apoptosis. **a** Analysis by western blot assay of PD-L1 and FKBP51s expression levels in D54MG cells, silenced for FKBP51s, using different siRNAs (siFKBP51s^UTR1^, siFKBP51s^UTR2^, and siFKBP51s) or PD-L1. The blot also shows caspase-7 levels recognized in its inactive (procaspase) and active forms. On the bottom of the panel, means and standard deviations of cell-death values of D54MG cells silenced for FKBP51s and PD-L1, are shown. Columns indicate the percentage of hypodiploid cells (*N* = 4). **b** Western blot assay of PD-L1 and FKBP51s levels in U251MG cells silenced for FKBP51s and PD-L1. On the bottom of the panel, means and standard deviations of cell-death values of U251MG cells silenced for FKBP51s and PD-L1, are shown. Columns indicate the percentage of hypodiploid cells (*N* = 4). **c** Graphical representation of means and standard deviations of cell-death values in PD-L1-depleted D54MG and U251MG cells. Twenty-four hours after transfection with PD-L1 siRNA, cells were treated with etoposide. They were then harvested after a further 24 h and analyzed for PI incorporation via flow cytometry. Columns indicate the percentage of hypodiploid cells (*N* = 3). **d** Flow-cytometric histograms of PD-L1 expression in D54MG and U251MG cells, cultured for 6 h with etoposide. On the right, a graphical representation is given of values (mean and standard deviation) from three different experiments. **e** Western blot assay of PD-L1 levels in GBM cells cultured with etoposide. **f** Measurement by qPCR of FKBP51s transcript level in cells treated or not treated with etoposide, for 6 h (*N* = 4). **g** Western blot assay of exogenous PD-L1 levels in D54MG and U251MG cells cultured in the absence or the presence of etoposide. **h** Graphical representation of means and standard deviations of cell-death values in PD-L1 D54MG and U251MG glioma cells. Twenty-four hours after transfection with EV or PD-L1-GFP, cells were treated with etoposide. They were then harvested after a further 24 h and analyzed for PI incorporation via flow cytometry. Columns indicate the percentage of hypodiploid cells. Each experiment was performed at least three times and in triplicate

We investigated the effect of PD-L1 silencing on GBM cell survival. To this end, we used two human GBM cell lines previously found to highly express PD-L1 and FKBP51s, namely, D54MG and U251MG cells^14^. For PD-L1 downmodulation, cells were treated with specific siRNAs targeting PD-L1 or its co-chaperone FKBP51s. Twenty-four hours after transfection, some of the cells were harvested for lysate preparation. After a further 24 h, the remaining cells were collected for cell-death measurements via PI incorporation. Figure 1a shows a western blot assay of lysates obtained from human D54MG cells treated with three different FKBP51s siRNAs and a specific PD-L1 siRNA (siPD-L1). Two of the three siRNAs were designed on the 3′-UTR (siFKBP51s^UTR1^ and siFKBP51s^UTR2^) and another (siFKBP51) on the coding region. The PD-L1 signals at ≈50 kDa are those of mature (glycosylated) forms and those under 37 kDa correspond to the naive protein^14^ (Fig. 1a). SiFKBP51s and siFKBP51s^UTR1^ appeared to downmodulate FKBP51s more efficiently than siFKBP51s^UTR2^. Expression of PD-L1 was also decreased by siFKBP51s and siFKBP51s^UTR1^, in comparison to the control cells (NSRNA or none). The procaspase-7 level was decreased by the same siRNAs, with a cleaved fragment at ≈20 kDa also observable, consistent with apoptosis activation (Fig. 1a). Measurement of hypodiploid cells confirmed that PD-L1 downmodulation, like FKBP51s silencing, produced cell death (Fig. 1a). The effect of different siRNAs on both FKBP51s and PD-L1, was also assessed in U251MG, as shown in the Supplementary Information (Fig. S1). Since siPD-L1 and siFKBP51s appeared to be the most effective for target downmodulation, these siRNAs were used in subsequent experiments. Human U251MG cells showed similar results to those obtained with D54MG cells (Fig. 1b). Both PD-L1 and FKBP51s silencing decreased PD-L1 expression levels, but only FKBP51s siRNA decreased the FKBP51s expression level. Activation of caspase-3 was registered in U251MG cells using flow cytometry (Supplementary Information, Fig. S2). We, then, investigated the effect of PD-L1 silencing on etoposide-induced cell death. Silencing of PD-L1 appeared to exert a cytotoxic effect similar to that of etoposide. However, combination of the two factors appeared to further increase cell death, in comparison with the single treatment. This result suggested that reduced levels of PD-L1 could act in concert with the chemotherapeutic compound to enhance its cytotoxicity (Fig. 1c). Using flow cytometry, we found that both cell lines, when cultured with etoposide for 6 h, had increased levels of PD-L1, compared to the same untreated cells (Fig. 1d). As expected, western blot analysis confirmed the increase in the mature PD-L1 signals at 50 kDa (Fig. 1e) and showed an increased expression of FKBP51s in etoposide-treated cells. These results suggested that etoposide induced *FKBP5* mRNA splicing, which was confirmed at the transcription level (Fig. 1f). Ectopic expression of PD-L1 (Fig. 1g), significantly reduced etoposide-induced cell death (Fig. 1h). Taken together, these results suggest that PD-L1 is a resistance factor for GBM cells. Further confirmation of the PD-L1 pro-survival effect was obtained with the other two GBM cell lines, namely, U87MG and SF767 (see Supplementary Information, Figs. S3 and S4 respectively). Interestingly, in SF767 cells, which show deficient PD-L1 levels^14^, PD-L1 silencing did not produce an apoptosis-enhancing effect, whereas PD-L1 ectopic expression reduced etoposide cytotoxicity (Supplementary Information, Fig. S4).

### FKBP51s promotes apoptosis resistance

**Fig. 2:**
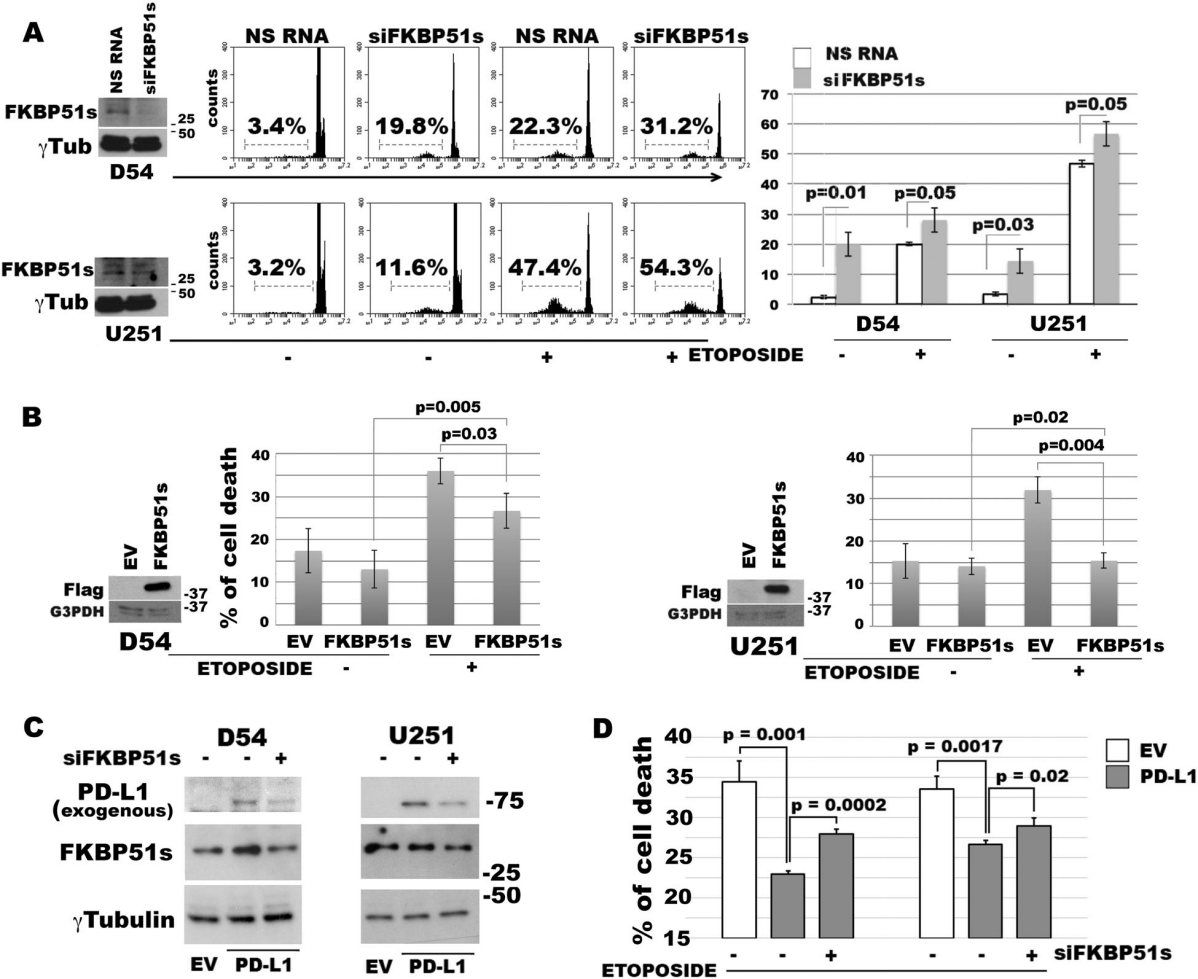
FKBP51s regulates glioma cell death. **a** Western blot assay of FKBP51s levels in D54MG and in U251MG silenced for FKBP51s. Cells were treated with FKBP51s siRNA or NSRNA for 24 h. Then, some of the cells were harvested for lysate preparation and some cells were treated with etoposide. After a further 24 h, cell death was measured. Representative flow-cytometric histograms of PI incorporation are shown along with graphical representations of means and standard deviations of cell-death values (*N* = 3). **b** Western blot assays of exogenous FKBP51s levels and graphical representations of means and standard deviations of cell-death values in D54MG and U251MG cells transfected with FKBP51s with EV- or FKBP51s-carrying plasmids (*N* = 3). Cells were treated with etoposide at 24 h from transfection. After a further 24 h, cell death was measured by flow cytometry (*N* = 3). **c** Western blot assays of the exogenous PD-L1-GFP level in D54MG and U251MG cells, silenced or not silenced for FKBP51s. Silencing of FKBP51s produced a decrease in PD-L1-GFP level in both cell lines. **d** D54MG and U251MG cells transfected with EV, PD-L1-GFP + NSRNA and PD-L1-GFP + FKBP51s siRNA, were treated with etoposide. After 24 h, cell death was measured using flow cytometry. FKBP51s depletion reduced the antiapoptotic effect of exogenous PD-L1. Each experiment was performed at least three times and in triplicate

Since FKBP51s deprivation reduces PD-L1 expression^14^, we investigated whether FKBP51s affected GBM cell death using RNA silencing. The effect of protein down-expression was assessed using western blot analysis (Fig. 2a). Cell death was assessed in the absence or the presence of etoposide, via PI incorporation. Figure 2a shows representative flow-cytometric histograms of hypodiploid events and a graphic representation of the means and standard deviations of cell-death values obtained in three independent experiments, each performed in triplicate. FKBP51s silencing in D54MG cells produced 20.0 ± 4.0% cell death, while treatment of cells with a non-silencing oligo (NSRNA) produced only 2.3 ± 0.5% cell death (*p* = 0.01) (Fig. 2a). Similarly, in etoposide-treated cells, FKBP51s siRNA and NSRNA produced 28.0 ± 4.0% and 20.0 ± 0.1% cell death respectively (*p* = 0.01) (Fig. 2a). FKBP51s-silenced U251MG produced an average of 14.3 ± 4.0% cell death, while NSRNA produced only 3.3 ± 0.5% cell death (*p* = 0.03) (Fig. 2a). In etoposide cultures, FKBP51s siRNA and NSRNA produced 55.0 ± 4.1% and 46.7 ± 1.1% cell death respectively (Fig. 2a) (*p* = 0.05). To confirm the role of FKBP51s in the resistance of GBM cells, we overexpressed FKBP51s and analyzed cell death using EV cells for comparison. In line with the FKBP51s knockdown sensitizing effect, we registered a significant decrease in etoposide-induced cell death in D54MG and U251MG cells transfected with FKBP51s vector, compared with EV cells (*p* = 0.03 and *p* = 0.004 for D54MG and U251MG, respectively) (Fig. 2b). To investigate whether PD-L1 requires FKBP51s to counteract chemotherapy-induced cell death, we overexpressed PD-L1 under conditions of FKBP51s depletion. To this end, we co-transfected PD-L1 and FKBP51s siRNA or NSRNA, in D54MG and U251MG cells (Fig. 2c), and measured cell death in etoposide cultures (Fig. 2d). The silencing of FKBP51s hampered the pro-survival effect of ectopic PD-L1 (Fig. 2d). The cell-death modulatory effect of FKBP51s was also assessed in U87MG cell lines (Supplementary Information, Fig. S5), as well as using SAFit2 (Supplementary Information, Fig. S6).

### PD-L1 expression increases with glioma aggressiveness and affects spheroid formation in culture

**Fig. 3:**
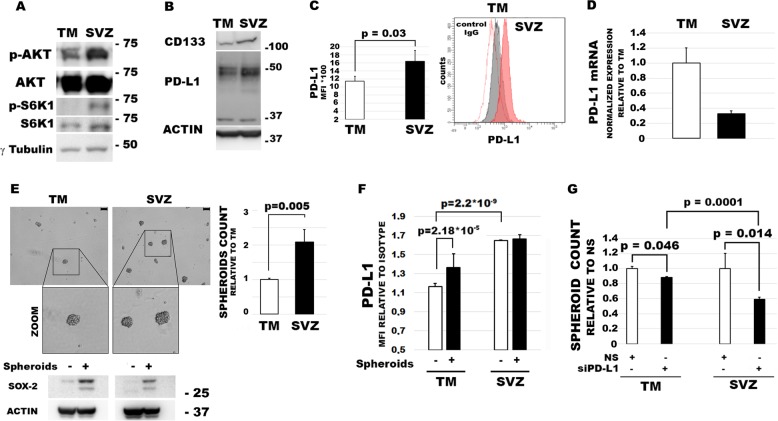
PD-L1 sustains GBM capacity to form spheroids. **a** Immunoblot of p-Akt and p-S6K1 in TM-GBM and SVZ-GBM cells. Levels of phospho-enzymes were higher in SVZ-GBM cells than in TM-GBM cells. **b** Expression of PD-L1 and CD133 in TM-GBM and SVZ-GBM cells. Levels of both proteins were higher in SVZ-GBM cells than in TM-GBM cells. **c** Flow cytometry measurements of PD-L1 expression in TM-GBM and SVZ-GBM cells. Graph columns represent mean fluorescence intensities with related *p*-values (*N* = 3). A representative histogram is shown in overlay. **d** RT-qPCR results for mRNA levels of PD-L1 in TM-GBM and SVZ-GBM cells. Graph columns represent normalized quantification relative to TM-GBM cells (*N* = 3). **e** Representative images of formed spheroids taken using an optical microscope and spheroid counts in TM-GBM and SVZ-GBM cultures (scale bar represents 100 μm). Graph columns represent spheroid numbers relative to TM-GBM cells (*N* = 3). A western blot of Sox-2 levels shows increased levels of the stemness marker in the spheroids (+), in comparison with adherent cells (−). **f** Quantification using flow cytometry of PD-L1 levels, in TM-GBM-formed spheroids and SVZ-GBM-formed spheroids. Graph columns represent MFI (*N* = 3). **g** Spheroid formation assay with TM-GBM and SVZ-GBM cells silenced or not silenced for PD-L1. Graph columns represent the relative spheroid numbers after a 4-day culture. NSRNA-treated cells were used as the reference sample. Each experiment was performed at least three times and in triplicate

The role of PD-L1 in the apoptosis resistance of GBM cells, together with its previous involvement in EMT^10^, supports the concept that this immune checkpoint is a hallmark of GBM malignancy. To address whether PD-L1 expression increased with the aggressiveness of GBM cells, we used two GBM cell lines previously established by Kroonen et al.^19^ from the tumor mass (TM-GBM) and the subventricular zone (SVZ-GBM) of an orthotopic GBM model. TM-GBM and SVZ-GBM cell lines, characterized by an increasing level of aggressiveness from TM to SVZ, were derived from the human U87MG cell line. SVZ-GBM cells are particularly rich in GBM cancer stem cells and have a high tumorigenic potential, as assessed by mouse reinjection^19^ or by their capacity to grow in spheres when cultured under non-adherent conditions^20^. We confirmed the high tumorigenicity of SVZ-GBM cells showing increased levels of p-Akt, along with its substrate p-S6K1 (Fig. 3a) and the stemness marker CD133 (Fig. 3b), in comparison to TM-GBM cells. SVZ-GBM also showed increased PD-L1 expression in contrast to TM-GBM cells, as assessed by western blot analysis (Fig. 3b) and flow cytometry (Fig. 3c). Surprisingly, PD-L1 mRNA levels appeared to be higher in the TM-GBM cells than in the SVZ-GBM cells (Fig. 3d). In line with Goffart et al.^20^, we found that SVZ-GBM cells have an increased capacity to form spheroids in comparison with TM-GBM cells (Fig. 3e). As expected, spheroids (+) expressed Sox-2 levels remarkably higher than those in the corresponding adherent cells (-) (Fig. 3e). Expression of PD-L1 resulted especially in an increase in spheroids formed by TM-GBM cells, in comparison with attached TM-GBM cells. Although only a slight increase in PD-L1 expression was observed in SVZ-GBM cells growing in spheres (Fig. 3f), the finding that PD-L1 silencing impaired the capacity to form spheroids in both TM- and SVZ-GBM cells (Fig. 3g) highlighted the importance of PD-L1 in spheroid formation. Taken together, our results indicate that PD-L1 is a necessary but not a sufficient condition for allowing the GBM cells to grow in spheres.

### Targeting of FKBP51s downregulates PD-L1 and hampers GBM malignancy

**Fig. 4:**
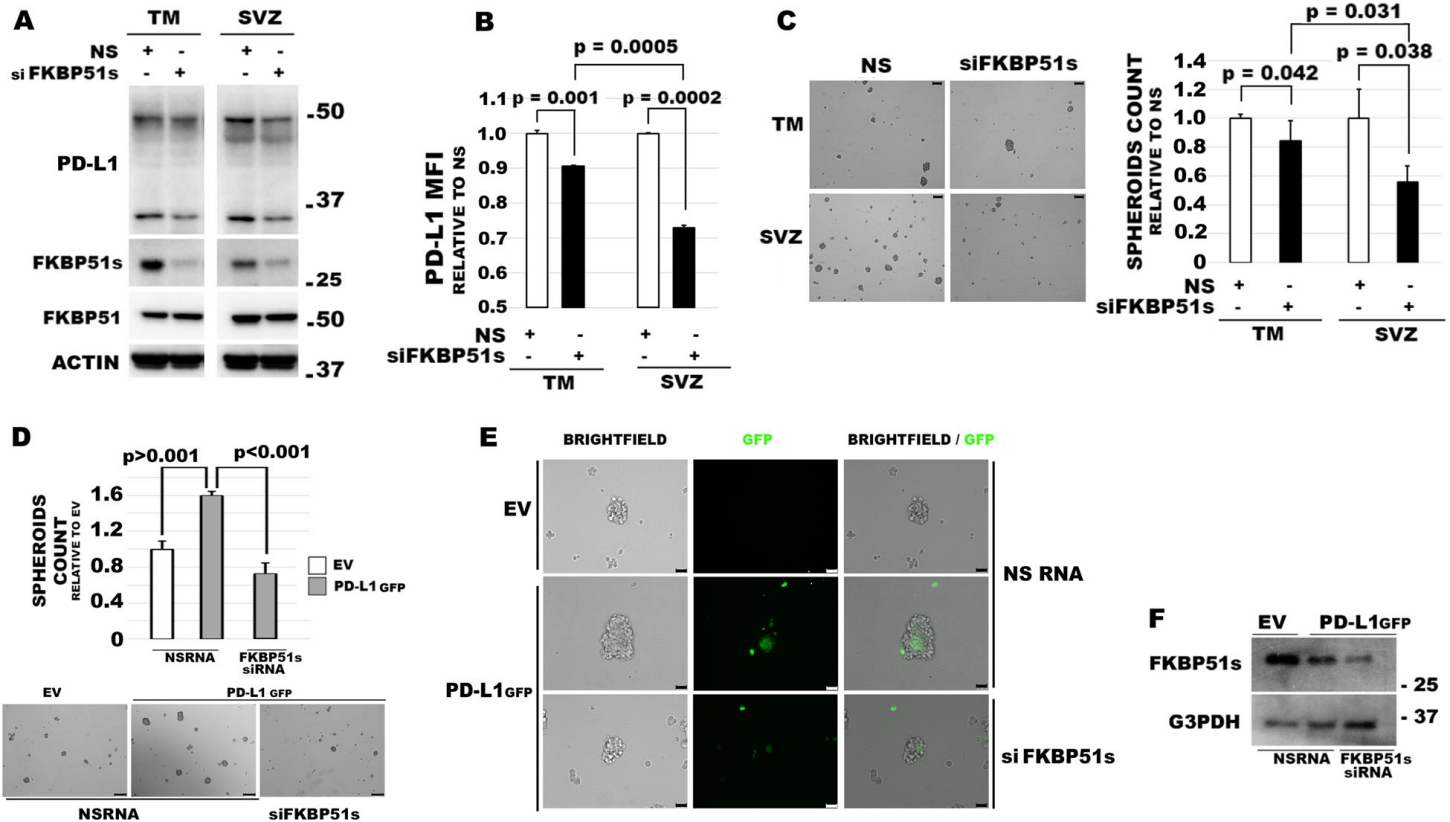
FKBP51s regulates PD-L1 expression and spheroid formation in TM-GBM and SVZ-GBM cells. **a** Immunoblot of PD-L1 and FKBP51s levels in cells silenced for FKBP51s. Canonical FKBP51 confirmed the specificity of the silencing. **b** Flow cytometry analysis of PD-L1 expression in cells, silenced or not silenced for FKBP51s. Graph columns represent MFI using non-silenced cells as the reference sample (*N* = 3). **c** Spheroid assay with cells from TM-GBM and SVZ-GBM cells, silenced or not silenced for FKBP51s. Representative images of formed spheroids are shown. Graph columns represent the relative spheroid numbers, after a 4-day culture, using NSRNA-treated cells as the reference sample (*N* = 3). **d** Spheroid assay with TM-GBM cells and SVZ-GBM cells transfected with EV, PD-L1-GFP+ NSRNA, and PD-L1-GFP + FKBP51s siRNA. FKBP51s depletion reduced the spheroid formation stimulated by exogenous PD-L1 (scale bar represents 250μm) (*N* = 3). **e** Fluorescent microscopy visualization of ectopic PD-L1-GFP in the spheroid assay (scale bar represents 50 μm). FKBP51s depletion reduced spheroid numbers and the extent of fluorescence. **f** Western blot assay of FKBP51s levels in FKBP51s-silenced spheroids. Each experiment was performed at least three times and in triplicate

**Fig. 5:**
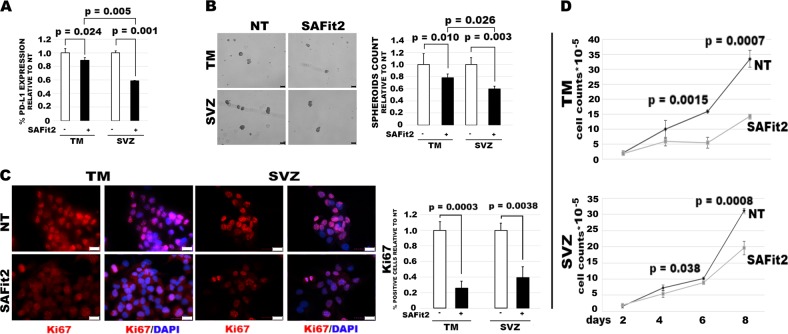
SAFit2 decreases PD-L1 expression and spheroid formation and impairs in vitro growth of TM-GBM and SVZ-GBM cells. **a** Cytometry analysis of PD-L1 expression in cells treated with SAFit2 for 12 h. Graph columns represent mean fluorescence intensities using cells with Dimethyl sulfoxide (DMSO) as the reference sample (*N* = 3). **b** Spheroid assay with cells treated with SAFit2 or the vehicle. Representative pictures of formed spheroids are shown along graph columns for the relative spheroid number measured after a 4-day culture. DMSO-treated cells were used as the reference sample (*N* = 3). **c** Ki67 expression measured by immunofluorescence in cells treated with SAFit2 or the vehicle (scale bar represents 25 μm). Graph columns represent the percentage of Ki67-positive cells, relative to the control (*N* = 6). **d** Kinetics of counts of TM-GBM and SVZ-GBM cells treated with SAFit2 or the vehicle. SAFit2 significantly reduced cell counts (*N* = 4)

**Fig. 6:**
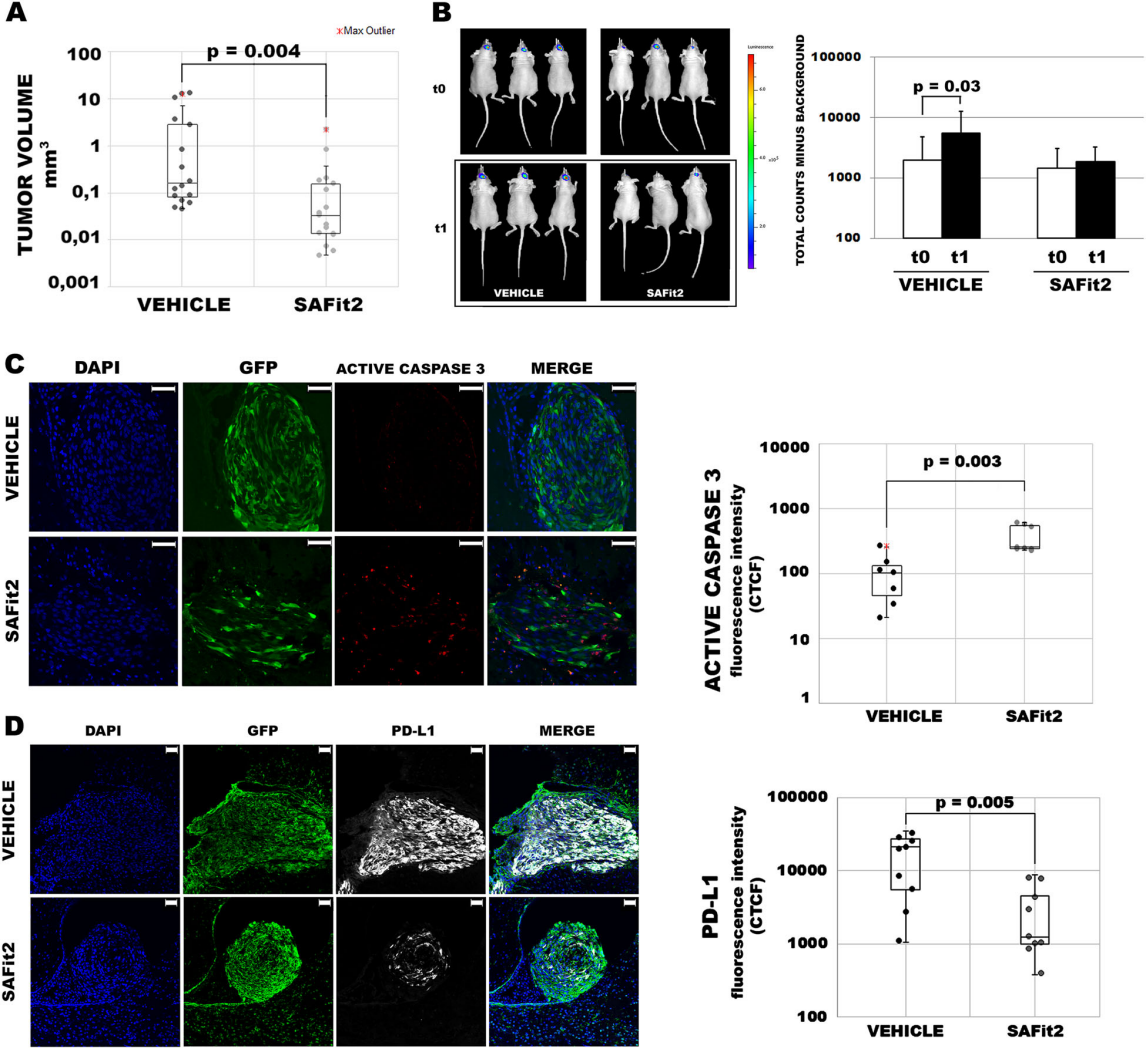
SAFit2 impairs GBM growth in vivo. **a** Graphic representation of tumor volumes formed in 16 mice treated daily with vehicle and 16 mice treated with SAFit2. **b** Luciferase assay, performed on mice treated or not treated with SAFit2, 2 weeks after in-brain injection with GFP+ luc+ U87MG cells. Three mice from each group are shown before starting the treatment and after 10 days (left). Graph columns represent mean values of signal intensities from the two groups, before and after treatment (right) (*N* = 16). **c** Expression of active caspase-3 by immunofluorescence on brain slices (scale bar represents 50 μm). Box plots represent the values of GFP+ cleaved Casp3+ cells measured using ImageJ software, from seven mice treated with vehicle and seven mice treated with SAFit2. **d** Expression of PD-L1 determined by immunofluorescence on brain slices (scale bar represents 50 μm). Box plots represent the values of GFP+ PD-L1+ cells measured using ImageJ software, from nine mice treated with vehicle and nine mice treated with SAFit2

Since the increased expression of PD-L1 in SVZ-GBM cells, in comparison to TM-GBM cells, appeared to be related to a post-transcriptional mechanism, we investigated the effect of FKBP51s silencing on the modulation of PD-L1 levels in both TM-GBM and SVZ-GBM cells. As shown in the western blot in Fig. 4a, the silencing of FKBP51s downmodulated PD-L1 levels in both cell lines. This result was confirmed using flow cytometry (Fig. 4b). FKBP51s downmodulation impaired spheroid formation in both TM-GBM and SVZ-GBM cells (Fig. 4c). Interestingly, SVZ-GBM appeared to be more sensitive than TM-GBM to the targeting of FKBP51s, according to the PD-L1 down-expression, which was more powerful in SVZ-GBM than in TM-GBM cells (Fig. 4b). FKBP51s silencing counteracted the effect of ectopic PD-L1-GFP in promoting spheroid formation (Fig. 4d, e). A western blot validated the impact of FKBP51s silencing on protein levels (Fig. 4f). We then investigated the effect of SAFit2 on PD-L1 expression and spheroid formation in TM-GBM and SVZ-GBM cells. PD-L1 expression, as assessed by flow cytometry, showed a significant decrease on the plasma membrane in SAFit2 cultures (Fig. 5a). Like FKBP51s siRNA, SAFit2 impaired spheroid formation in both TM-GBM and SVZ-GBM cells (Fig. 5b). To address whether SAFit2 could affect tumor growth, we assessed Ki67 expression and cell counts in TM-GBM and SVZ-GBM cell cultures (Fig. 5c, d). On SAFit2 treatment, both cell lines showed a significant decrease in Ki67 expression (Fig. 5c) and cell counts (Fig. 5d). To address the in vivo effect of SAFit2, U87MG cells (carrying GFP and the luciferase reporter gene) were injected into the right striatum of 32 nude mouse brains. Two weeks later, 16 mice were treated daily with SAFit2, while 16 mice were treated intraperitoneally with a vehicle designed to pass through the brain-blood barrier, via intraperitoneal injection. After 2 weeks of treatment (corresponding to 4 weeks from cell xenotransplantation) the mice were sacrificed, and the brains obtained. A reduced tumor mass was calculated in mice treated with SAFit2 compared to tumors from mice treated with the vehicle (Fig. 6a). The result was confirmed by a reduced luminescence intensity recorded in the living mice before the sacrifice (Fig. 6b). The reduced tumor growth was accompanied by the presence of active caspase-3 in xenografts from the SAFit2-treated mice (Fig. 6c). In accordance with in vitro findings, SAFit2 reduced tumor PD-L1 expression (Fig. 6d). Finally, in-brain slices, the percentage of tumor cells found to be positive for the expression of the mesenchymal marker vimentin, was still reduced in mice treated with SAFit2 (Supplementary Information, Fig. S6).

## Discussion

A growing body of literature supports the concept that PD-L1 has intrinsic pro-tumoral aspects that are independent of its immunoinhibitory functions^6^. Moreover, several recent studies report that expression levels of PD-L1 positively correlate with glioma grades. More precisely, in GBM, these levels are much higher than in grade II and grade III gliomas^6^.

In this paper, consistent with the finding of Azuma et al.^8^ that PD-L1 is a ubiquitous antiapoptotic receptor, we show that PD-L1 is an essential element in GBM resistance to cell death. A previous paper also reported that PD-L1 overexpression, as a result of the low miR-34a level in U87 glioma cells, induced paclitaxel resistance^21^. In the present paper, we confirm the resistance effect of PD-L1 in four different glioma cell lines, namely, U87MG, D54MG, U251MG and SF767. Moreover, in accordance with Zheng et al.^9^, who demonstrated a role for PD-L1 in sustaining the self-renewal capability of malignant melanoma-initiating cells, we show that PD-L1 expression is increased in GBM cancer-initiating cells and has a supporting role in spheroid formation. Finally, in accordance with Qiu et al.^10^, who demonstrated the promoting role of PD-L1 in GBM-cell migration, we found increased expression of PD-L1 in GBM cells that had migrated to the subventricular zone of the brain, compared with PD-L1 expression in the nigrostriatal tumor mass. When the levels of PD-L1 were downmodulated by FKBP51s silencing, GBM-cell sensitivity to both spontaneous and chemotherapy-induced death was increased. Moreover, when the levels of PD-L1 were upregulated by an ectopic expression, a pro-survival effect on GBM cells was observed. This outcome was counteracted by FKBP51s silencing, which is consistent with the essential role for this co-chaperone in PD-L1 expression^14^. FKBP51s appeared to significantly affect the spheroid formation ability of GBM cancer-initiating cells, along with PD-L1. Collectively, these findings, in addition to supporting the pro-oncogenic role of PD-L1 in glioma, open up the possibility of targeting PD-L1 by acting on its co-chaperone FKBP51s. SAfit2 is, so far, the most well-characterized FKBP51 ligand available^22^. After 2 weeks of intraperitoneal treatment with SAFit2, assessment of GFP + luc + tumor volumes in-brain slices showed a significant decrease in SAFit2-treated mice, in comparison with vehicle-treated control mice. Consistent with this finding, reduced luminescence intensity was registered in SAFit2-treated tumors in living mice before sacrifice. The presence of active caspase-3 in tumor xenografts from SAFit2-treated animals suggested that the reduction in tumor volumes can be ascribed, at least in part, to apoptosis induction. A decreased percentage of tumor cells expressing PD-L1 and vimentin, according to histological examinations, suggested that in treated mice the tumor had a reduced aggressiveness.

In conclusion, in line with a previous study demonstrating that in vivo knockdown of PD-L1 in nude mice completely abolished GBM xenograft formation^10^, our finding shows that manipulation of PD-L1, through chemical inhibition of its co-chaperone FKBP51s, activates apoptosis and mitigates the aggressive features of GBM xenografts in nude mice. Our study contributes some information to the emerging concept that there is a strict interplay between cancer immunoediting and resistance and suggests that SAFit2 may be a beneficial neoadjuvant strategy for the management of this tumor.

## Methods and materials

### Cell culture and transfection

Human glioma cell lines D54MG, U251MG, SF767MG were obtained and cultured as described^14^. U87MG from American Type Culture Collection (ATCC) were cultured in Dulbecco’s Modified Eagle Medium (DMEM) containing 10% FBS, 200 mM glutamine, and 100 U/mL penicillin–streptomycin. TM-GBM cells and SVZ-GBM cells were U87MG cells obtained by microdissection of xenografted mice brain^19^, from, respectively, the tumor mass and the subventricular zone, and seeded in cultures^19^. For FKBP51s and PD-L1 knockin and knockdown, cells were transfected using the K2 Transfection System (Biontex, Munich, Germany), as previously described^14^ and in accordance with the manufacturer’s recommendations. For overexpressing FKBP51s and PD-L1 a True-ORF-Myc-DDK-tagged and a True-ORF-GFP-tagged expression vectors were used (OriGene Technologies, Rockville, MD, USA), which carried the complementary DNA (cDNA) of the human FKBP5 transcript variant 4 and CD274, respectively. Control cells were transfected with the related empty vector (EV). FKBP51s silencing was performed using short-interfering oligoribonucleotides. The siFKBP51 was a mix of two siRNAs designed on the coding region; siFKBP51s^UTR1^and FKBP51s^UTR2^ were designed on 3′-UTR^14^. The non-silencing RNA (NSRNA) and PD-L1 siRNA were from Qiagen (siPD-L1) (Valencia, CA, USA) or Novus Biologicals (siPD-L1.2) (Littleton, CO). U87MG cells used for xenograft implantation were previously transduced with lentiviral vectors allowing the dual expression of eGFP and luciferase as previously described^20^. In experiments with etoposide, the drug was added 24 h after transfection at a concentration of 20 μM. For experiments with SAFit2^22^, which was provided by Prof. Felix Hausch laboratory, a stock solution (60 μM in DMSO) was used at 1:1000 dilution in DMEM-F12 media; for control cells, DMSO has diluted accordingly.

### Western blot

Whole-cell lysates were homogenized in modified RIPA buffer^14^ and assayed by immunoblot as previously described^14^. The primary antibodies against FKBP51 (rabbit polyclonal; Novus Biological), FKBP51s^13^ and CD274/PD-L1 (rabbit polyclonal; Novus Biological) were used diluted 1:2500. CD133 (rabbit polyclonal; Abcam; Cambridge, UK) was used diluted 1:1000. A further antibody Pdcd-1L1 (rabbit polyclonal, Santa Cruz Biotechnology; Santa Cruz, CA, USA) was used for PD-L1 detection at the 1:1000 dilution. Antibody against M2-Flag, caspase-7 and γ-Tubulin (mouse monoclonal; Sigma-Aldrich, St. Louis, MO, USA) were used diluted 1:5000. Anti β-Actin-Peroxidase (mouse monoclonal; Sigma-Adrich) was used diluted 1:10000. Anti-phospho-Akt (Ser473), Akt1/2/3, phosphor-S6 kinase, G3PDH and Sox-2 (rabbit monoclonal; Cell Signaling, Danvers, MA, USA) were used diluted 1:1000. Anti-p70S6 kinase (rabbit polyclonal; Santa Cruz) was used diluted 1:500.

### Flow cytometry

Expression of PD-L1 was assessed using anti-B7H1-phycoerythrin (PE) (R&D Systems, Minneapolis, MN, USA) at a concentration of 0.05 μg/ml. A PE-conjugated control Ig isotype was used to assess non-specific binding. Briefly, cells were harvested and incubated with the antibodies mentioned above for 30 min in the dark at 4 °C, washed and then analyzed with a BD Accuri™ C6 Cytometer (BD Biosciences, New Jersey, USA). Cleaved caspase-3 immunostaining was performed as previously described^23^. Apoptosis was assessed by two methods: propidium iodide (PI) incorporation to analyze the DNA content in permeabilized cells^24^, and the annexin V-binding assay in double staining with PI, according to Vermes et al.^25^. Cells were analyzed with a BD Accuri™ C6 Cytometer (BD, Becton Dickinson).

### Microscopy

Before the immunostaining, cells were seeded for 3 h on coverslips coated with polyornithine (0.1 mg/ml), then, after washing with PBS the cells were fixed in 4% paraformaldehyde (PFA) for 15 min. Brain coronal sections or cells on coverslips were permeabilized and unspecific binding sites were blocked for an hour at room temperature using a 10% donkey serum and 0.2% Triton X-100 PBS solution. Tissue sections or coverslipped cells were then incubated overnight at 4 °C with primary antibodies diluted in PBS containing 0.1% of donkey serum and 0.1% of Triton X-100, followed by tetramethylrhodamine (TRITC)-, fluorescein isothiocyanate (FITC)-conjugated, or cyanine 5 (Cy5) secondary antibodies (Jackson Immunoresearch Laboratories; Cambridge, UK) diluted 1:500. Anti Ki-67 (BD Biosciences, Becton Dickinson) was used diluted 1:500. Anti-Vimentin (rabbit monoclonal IgG, Cell Signaling, Danvers, MA, USA) was used diluted 1:400. Anti-PD-L1 (mouse monoclonal, Abcam; Cambridge, UK) was used diluted 1:200. Anti-Active Caspase-3 (Affinity-purified rabbit IgG, Promega, Madison, Wisconsin, USA) was diluted 1:250. Anti-GFP (chicken polyclonal, Abcam; Cambridge, UK) was used diluted 1:500. Counterstaining with DAPI (D9542, Sigma-Aldrich, St. Louis, MO, USA) was performed and slides coverslipped with Fluoromount-G (00–4958–02, Invitrogen, Carlsbad, CA, USA). Images were acquired with OLYMPUS FV1000 confocal microscopy. For PD-L1 and active caspase-3 expression, fluorescence intensity was quantified according to “corrected total cryosection fluorescence” (CTCF)^26^. Quantification of vimentin expression in-brain slices was performed as followed: mean fluorescence intensity (MFI) was calculated for each GFP+ cell stained with anti-vimentin along with GFP+ cell stained with control antibody. Positivity was assigned when vimentin MFI was at least four-fold higher than control MFI. When required, the brightness, contrast, and color balance of the images were adjusted in Photoshop CS2 (Adobe Systems, San Jose, California, USA). This adjustment was applied to every pixel in each image.

### Quantitative PCR (qPCR)

Total RNA was extracted from cells by Trizol (Invitrogen, Carlsbad, CA, USA). Each RNA was used for cDNA synthesis with iScript Reverse Transcription (Bio-Rad, CA, USA). Relative gene expression was quantified by qPCR with 2–ΔCt comparative method^27^, using the SsoAdvancedTM SYBR Green Supermix (Bio-Rad) and specific qPCR primers. Amplification of FKBP51s was performed as previously described^13^. Oligo sequences used for PD-L1 mRNA quantitation along with co-amplified housekeeping genes are:

Fw-PD-L1 5′-GCTTTTCAATGTGACCAGCA-3′

Rev-PD-L1 5′-TGGCTCCCAGCCTTACCAAG-3′

Fw-18S 5′-CGATGCGGCGGCGTTATTC-3′

Rev-18S 5′-TCTGTCAATCCTGTCCGTGTCC-3′

Fw-GAPDH 5′-GGACTCATGACCACAGTCCAT-3′

Rev-GAPDH 5′-GTTCAGCTCAGGGATGACCTT-3′

Fw-β-Actin 5′-CGAGGCCCAGAGCAAGAGAG-3′

Rev-β-Actin 5′-CGGTTGGCCTTAGGGTTCAG-3′

### Spheroid formation assay

TM- and SVZ-GBM cells were cultured in DMEM/F12 serum-free medium containing B-27-Supplement without vitamin A (Life Technologies; Carlsbad, CA, USA) and supplemented with recombinant epidermal growth factor and fibroblast growth factor 2 (EGF, 20 ng/mL and FGF-2, 10 ng/mL; Preprotech, London, UK). After 4 days in culture, the number of spheroids was counted using an optical microscope.

### Proliferation assay

TM- and SVZ-GBM cells were daily treated with 60 nM SAFit2 or opportunely diluted DMSO. Every 2 days, cells were harvested, and cell number was estimated by the Countess II Automated Cell Counter (Life Technologies). After a 4-day treatment, cells were seeded on a coverslip and processed for Ki67 immunostaining as previously described.

### Animal studies

P40 female immune-deficient mice (Crl:NU-Foxn1nu) were obtained from Charles River Laboratories (Charles River Laboratories®, Wilmington, UK) and handled according to the Animal Ethical Committee of the University of Liège. All animals were cared for in accordance with the Declaration of Helsinki and the guidelines of the Belgium Ministry of Agriculture, in agreement with the European Commission Laboratory Animal Care and Use Regulation (86/609/CEE, CE of J nL358, 18 December 1986). For intracranial transplantation, mice were anesthetized with an intraperitoneal injection of ketamine (50 mg/mL, Pfizer, Bruxelles, Belgium) and xylazine (Sedativum 2%, Bayer, Bruxelles, Belgium) solution (V/V). The cranium was exposed and a small hole was drilled 2.5 mm lateral and 0.5 mm anterior to the bregma with a size 34 Dremel Inverted Cone Burr. Mice were positioned in a stereotactic frame and 50000 GFP+ luc+ U87MG cells in 2 µl PBS were injected into the right striatum through a 27-gauge needle over 1 min at 3 mm below the dura mater. The incision was closed with 3 M Vetbond Tissue Adhesive (Fisher Scientific, Hampton, NH, USA). For mice treatment, with SAFit2, this was performed as previously described^22^. Before obtaining brains, mice were anaesthetized with an injection of Nembutal (Pentobarbital 60 mg/mL; Ceva Sante Animal, Libourne, France) followed by an intracardiac perfusion with a NaCl 0.9% solution (VWR International, Radnor, PA, USA) plus 4% PFA at 4 °C (4,3 g/L NaOH, 40 g/L PFA, 18.8 g/L NaH2PO4). Then after, brains were collected, postfixed in 4% PFA and cryoprotected overnight in a 20% PBS/sucrose solution. Brains were frozen at −20 °C in a 2-methylbutane solution (Sigma-Adrich) and cut into 14 μm thick coronal sections using a cryostat. The tumor volume was calculated on GFP+ areas using ImageJ and, according to Kim et al.^28^, the formula (Volume = 0.5 × length × width × height). For bioluminescence imaging: immunodeficient nude mice bearing intracranial xenografts were injected intraperitoneally with d-luciferin (150 mg/kg, Sigma-Adrich). After anesthesia using 2.5% isoflurane, mice were imaged with a charge-coupled device camera-based bioluminescence imaging system (IVIS 50, Xenogen; exposure time 1–30 s, binning 8, field of view 12, f/stop 1, open filter).

### Statistical analysis

Student’s *t*-test was used to analyze differences between means of values. A *p*-value of ≤0.05 was considered statistically significant.

## Supplementary information

Supplementary figures to the manuscript

## References

[1] D’Arrigo, P. et al. Manipulation of the immune system for cancer defeat: a focus on the T cell inhibitory checkpoint molecules. *Curr. Med. Chem*. (2018). https://doi.org/0.2174/0929867325666181106114421, (Epub ahead of print). 10.2174/0929867325666181106114421 30398102

[2] Wainwright DA (2014). Durable therapeutic efficacy utilizing combinatorial blockade against IDO, CTLA-4, and PD-L1 in mice with brain tumors. Clin. Cancer Res..

[3] Berghoff AS (2015). Programmed death ligand 1 expression and tumor-infiltrating lymphocytes in glioblastoma. Neuro. Oncol..

[4] Nduom EK (2016). PD-L1 expression and prognostic impact in glioblastoma. Neuro Oncol..

[5] Parry RV (2005). CTLA-4 and PD-1 receptors inhibit T-cell activation by distinct mechanisms. Mol. Cell. Biol..

[6] Chen RQ, Liu F, Qiu XY, Chen XQ (2018). The prognostic and therapeutic value of pd-l1 in glioma. Front. Pharmacol..

[7] Butte MJ, Keir ME, Phamduy TB, Sharpe AH, Freeman GJ (2007). Programmed death-1 ligand 1 interacts specifically with the B7-1 costimulatory molecule to inhibit T cell responses. Immunity.

[8] Azuma T (2008). B7-H1 is a ubiquitous antiapoptotic receptor on cancer cells. Blood.

[9] Zheng F (2017). PD-L1 promotes self-renewal and tumorigenicity of malignant melanoma initiating cells. Biomed. Res. Int..

[10] Qiu XY (2018). PD-L1 confers glioblastoma multiforme malignancy via Ras binding and Ras/Erk/EMT activation. Biochim. Biophys. Acta Mol. Basis Dis..

[11] Parsa AT (2007). Loss of tumor suppressor PTEN function increases B7-H1 expression and immunoresistance in glioma. Nat. Med..

[12] Li CW (2016). Glycosylation and stabilization of programmed death ligand-1 suppresses T-cell activity. Nat. Commun..

[13] Romano S (2015). Immunomodulatory pathways regulate expression of a spliced FKBP51 isoform in lymphocytes of melanoma patients. Pigment Cell Melanoma Res..

[14] D’Arrigo P (2017). A regulatory role for the co-chaperone FKBP51s in PD-L1 expression in glioma. Oncotarget.

[15] Hsu JM, Li CW, Lai YJ, Hung MC (2018). Posttranslational modifications of PD-L1 and their applications in cancer therapy. Cancer Res..

[16] Jiang W (2008). FK506 binding protein mediates glioma cell growth and sensitivity to rapamycin treatment by regulating NF-kappaB signaling pathway. Neoplasia.

[17] Romano S (2011). The emerging role of large immunophilin FK506 binding protein 51 in cancer. Curr. Med. Chem..

[18] Romano S, D’Angelillo A, Staibano S, Ilardi G, Romano MF (2010). FK506-binding protein 51 is a possible novel tumoral marker. Cell Death Dis..

[19] Kroonen J (2011). Human glioblastoma-initiating cells invade specifically the subventricular zones and olfactory bulbs of mice after striatal injection. Int. J. Cancer.

[20] Goffart N (2015). Adult mouse subventricular zones stimulate glioblastoma stem cells specific invasion through CXCL12/CXCR4 signaling. Neuro Oncol..

[21] Wang Y, Wang L (2017). miR-34a attenuates glioma cells progression and chemoresistance via targeting PD-L1. Biotechno. Lett..

[22] Gaali S (2015). Selective inhibitors of the FK506-binding protein 51 by induced fit. Nat. Chem. Biol..

[23] Giordano A (2016). Tirofiban counteracts endothelial cell apoptosis through the VEGF/VEGFR2/pAkt axis. Vasc. Pharmacol..

[24] Romano S (2010). Role of FK506 binding protein 51 [FKBP51] in the control of apoptosis of irradiated melanoma cells. Cell Death Differ..

[25] Vermes I, Haanen C, Steffens-Nakken H, Reutelingsperger C (1995). A novel assay for apoptosis. Flow cytometric detection of phosphatidylserine expression on early apoptotic cells using fluorescein labelled Annexin V. J. Immunol. Methods.

[26] Lin D (2017). Tenomodulin is essential for prevention of adipocyte accumulation and fibrovascular scar formation during early tendon healing. Cell Death Dis..

[27] Schmittgen TD, Livak KJ (2008). Analyzing real-time PCR data by the comparative C(T) method. Nat. Protoc..

[28] Kim W (2015). Real-time imaging of glioblastoma using bioluminescence in a U-87 MG xenograft model mouse. J. Korean Soc. Appl. Biol. Chem..

