# Supplementary material for: The splicing FK506-binding protein-51 isoform plays a role in glioblastoma resistance through programmed cell death ligand-1 expression regulation
Source: Cell Death Discov. 2019 Sep 24;5:137. doi: 10.1038/s41420-019-0216-0 (PMC6760221; doi:10.1038/s41420-019-0216-0)
Supplement: Supplementary file 1 — Supplementary figures to the manuscript [file 41420_2019_216_MOESM1_ESM.pdf]

Supplementary Information

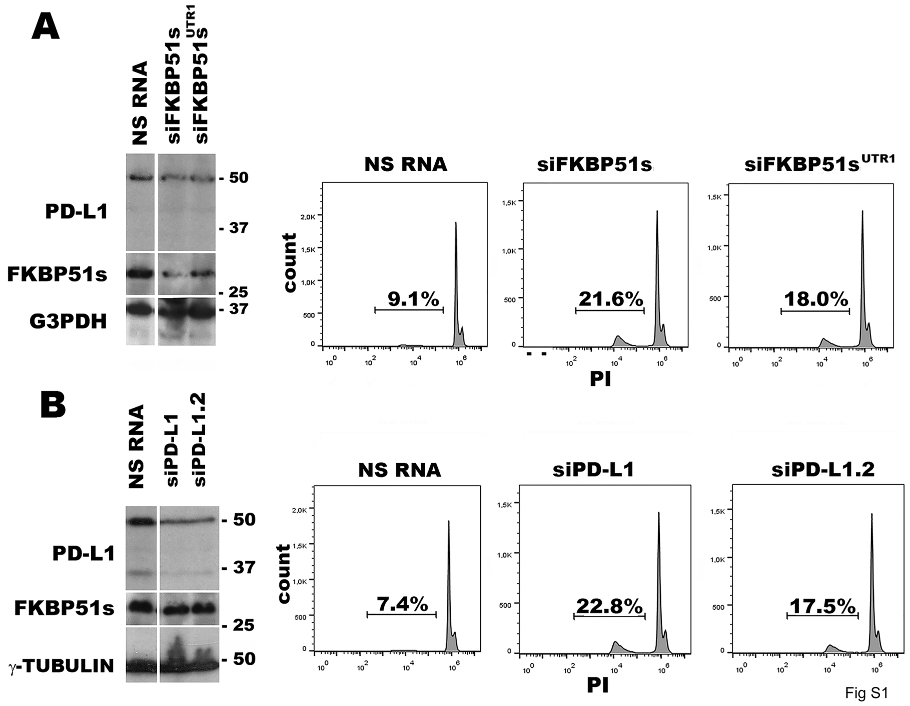

Fig. S1. Silencing of FKBP51s and PD-L1 expression.

(**A**) Western blot assay of PD-L1 and FKBP51s expression in U251MG cells silenced for FKBP51s via two different siRNAs. Representative flow-cytometry histograms of hypodiploid cells after silencing, are shown. The effect of siFKBP51s on FKBP51s expression appeared to be higher than that of siFKBP51s^{UTR1}. In addition, siFKBP51s also produced a PD-L1 downmodulating effect and U251MG cell death to an increased extent, compared with siFKBP51s^{UTR1}. (**B**) Western blot assay of PD-L1 and FKBP51s expression in U251MG cells silenced for PD-L1 via two different siRNAs. Representative flow-cytometry histograms of hypodiploid cells after silencing, are shown . The effect of siPD-L1 on PD-L1 expression appeared to be higher than that of siPD-L1.2. Furthermore, siPD-L1 also produced U251MG cell death to an increased extent, compared with siPD-L1.2.

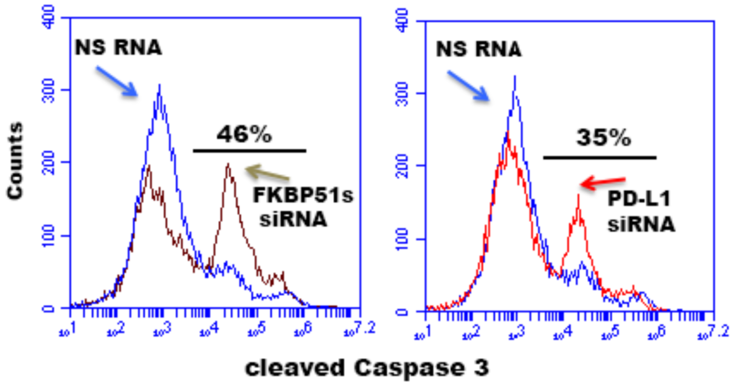

Fig S2

Fig. S2. PD-L1 and FKBP51s silencing promotes caspase activation.

Flow-cytometric histograms of active caspase-3 from U251 cells silenced for FKBP51s or PD-L1. Cells were harvested 24 hours after transfection. Bars indicate the percentage of active caspase-3 in FKBP51s-silenced cells (left, brown histogram) and PD-L1-silenced cells (right, red histogram). NSRNA is shown in the blue histogram.

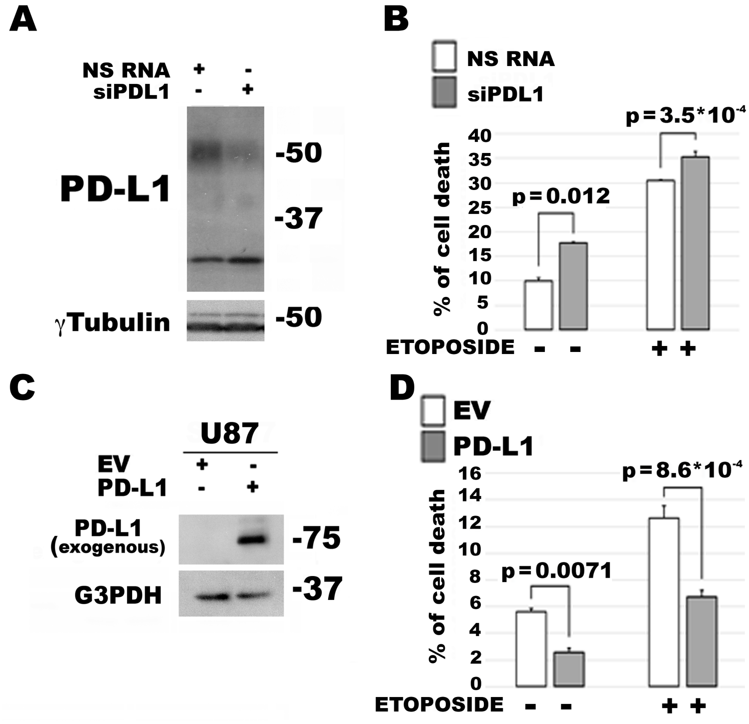

Fig S3

Fig. S3. PD-L1 expression regulates cell death in U87MG.

(**A**) Western blot assay of PD-L1 expression in U87MG cells silenced for PD-L1 or treated with a NSRNA. (**B**) Graph bars representing means and standard deviations of cell death in PD-L1-silenced U87MG cells in comparison with NSRNA-transfected cells, treated or not treated with 20μM etoposide. (**C**) Western blot assay of exogenous PD-L1 expression in U87MG cells transfected with a plasmid containing cDNA of PD-L1 tagged with the GFP gene, compared with cells transfected with a void vector (EV). (**D**) Graphic representation of means and standard deviations of apoptosis in U87MG cells overexpressing PD-L1 compared with EV cells, treated or not treated with 20μM etoposide.

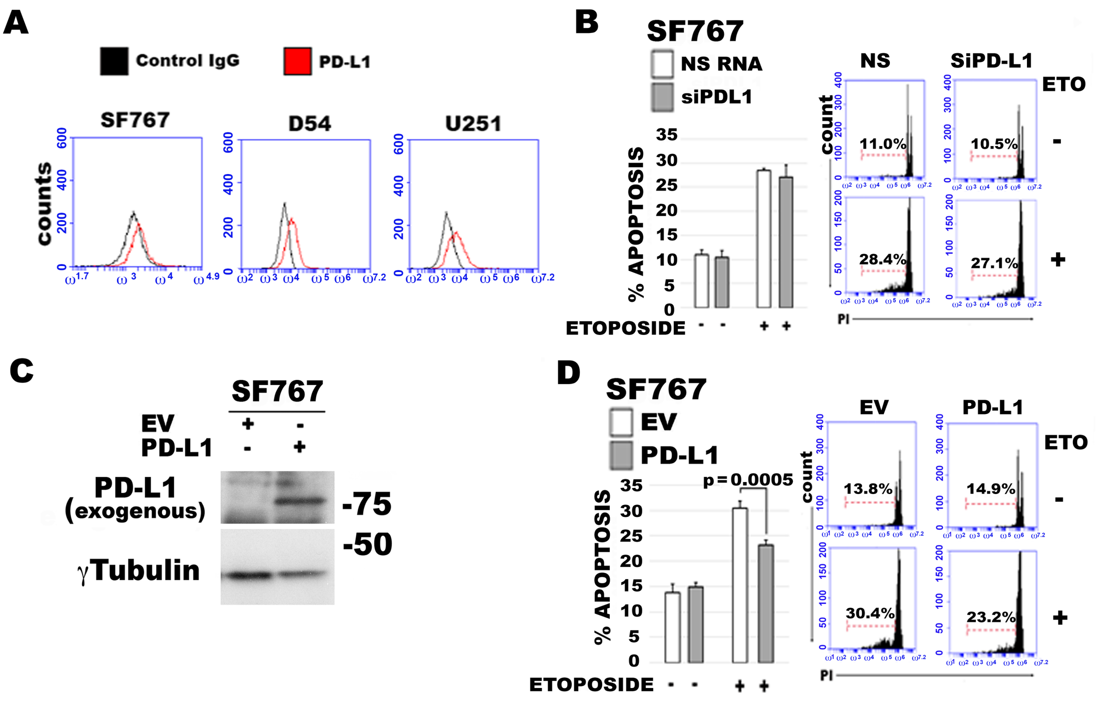

Fig S4

Fig. S4. Effect of ectopic PD-L1 on SF767 cell death.

(A) Flow-cytometric histograms of PD-L1 expression in SF767, compared with expression in D54MG and U251MG cells. The histograms of PD-L1 expression are shown with their related IgG-negative controls. (**B**) Graphic representation of means and standard deviations of apoptosis in SF767 cells silenced for PD-L1, compared with NSRNA-transfected cells, treated or not treated with 20µM etoposide. Representative flow-cytometric histograms of cell death are also shown. (**C**) Western blot assay of exogenous PD-L1 expression in SF767 cells transfected with a plasmid containing cDNA of PD-L1 tagged with the GFP gene, compared with cells transfected with a void vector (EV). (**D**) Graphic representation of means and standard deviations of cell-death values in cultures from SF767 overexpressing PD-L1 in comparison with EV cells, treated or not treated with 20µM etoposide. Representative flow-cytometric histograms of cell death are also shown.

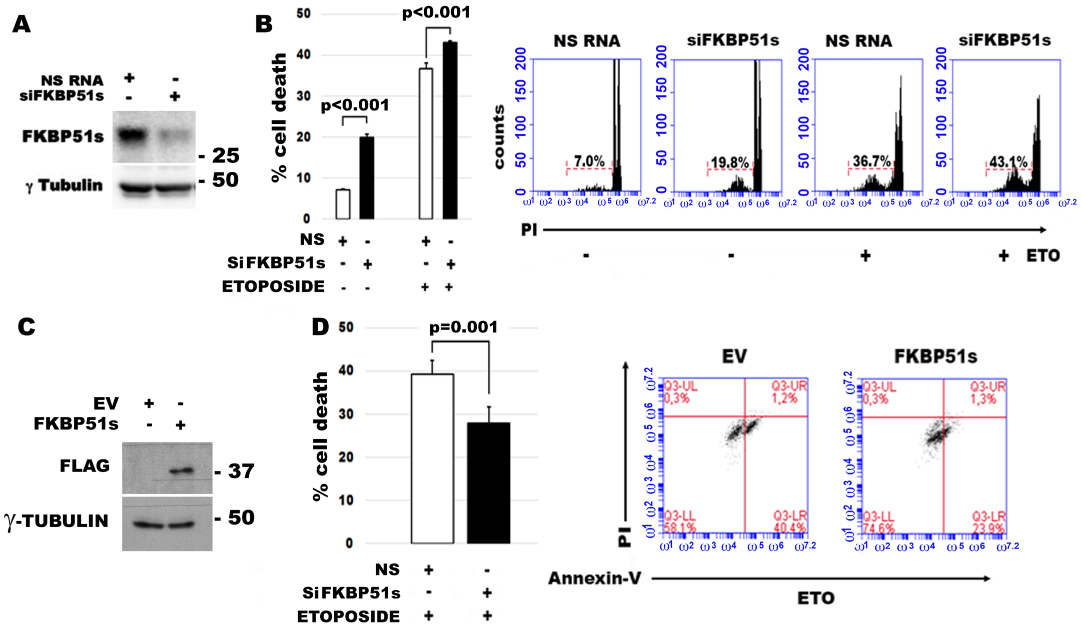

Fig S 5

Fig. S5. FKBP51s regulates U87MG cell death.

(A) Western blot assay of FKBP51s expression in U87MG cells silenced for FKBP51s in comparison with NSRNA-transfected cells. (**B**) Graphic representation of means and standard deviations of cell death in U87MG cells silenced for FKBP51s, compared with NSRNA-transfected cells, treated or not treated with 20μM etoposide after transfection. Representative flow-cytometric histograms of DNA content via PI incorporation are shown. (**C**) Western blot analysis of exogenous FKBP51s expression in U87MG. (**D**) Graphic representation of means and standard deviations of cell death in U87MG overexpressing FKBP51s, compared with EV cells treated with 20μM etoposide. Cell death was measured using Annexin V/PI staining. Representative flow-cytometric histograms are shown.

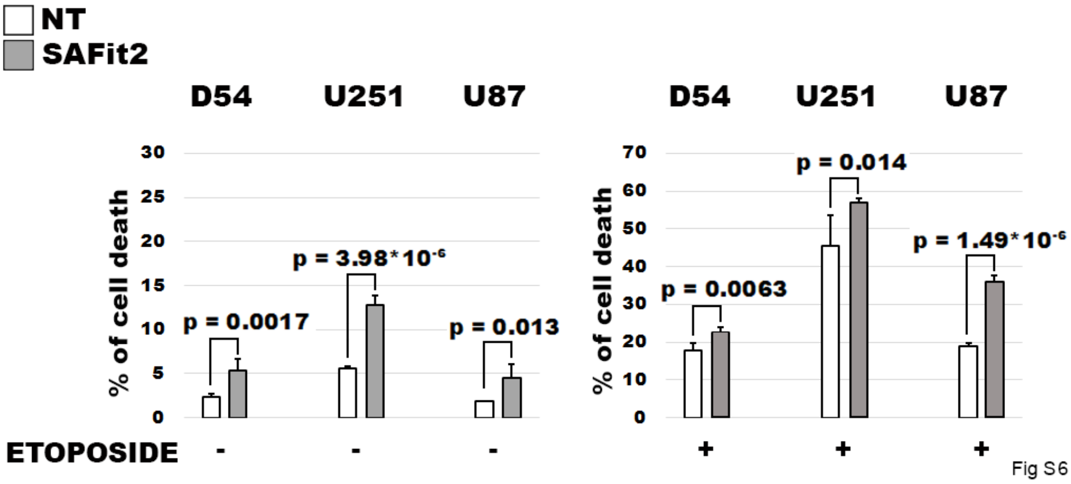

Fig. S6. SAFit2 sensitizes glioma cells to apoptosis induced by etoposide. Graphic representation of means and standard deviations of apoptosis in D54MG, U251MG and U87MG, treated or not treated with 20µM etoposide. Cells were pretreated overnight with 60nM SAFit2 or vehicle, before treatment with etoposide.
